# Premenopausal Bilateral Oophorectomy Effects on Clinical and Real-World Physical Function Measures

**DOI:** 10.1093/geroni/igab046.640

**Published:** 2021-12-17

**Authors:** Emma Fortune, Omid Jahanian, Melissa Morrow, Virginia Miller, Michelle Mielke

**Affiliations:** Mayo Clinic, Rochester, Minnesota, United States

## Abstract

Women with premenopausal bilateral oophorectomy (PBO) are at increased risk for physical function (PF) declines. This study investigated the relationships of field-based physical activity measures with clinical PF and strength parameters in post-menopausal women with and without PBO. Women with (n=21; age=64±4 years; BMI=32±8 kg.m-2) and without (n=15; age=67±6 years; BMI=28±6 kg.m-2) PBO performed PF and strength tests (walking speed, distance walked, short physical performance battery (SPBB), leg and chest strength), and wore ankle accelerometers for 7 days (daily step count and loading index [the cumulative sum of each step’s skeletal loading]). Age, BMI, step count and loading index were entered into stepwise multiple regression to identify significant predictors of PF and strength parameters. Step count was a predictor of SPBB score in both groups. In women without PBO, step count was a predictor of walking speed; loading index was a predictor of leg strength; step count and loading index were predictors of distance; and step count and age were predictors of chest strength. For PBO women, loading index and BMI were predictors of walking speed and distance; BMI was a predictor of leg strength; and there were no predictors of chest strength. These data suggest while field-based physical activity was strongly and positively associated with clinical PF and strength measures for women without PBO, BMI was a dominant negative factor for PF in women with PBO. Future work will include a larger sample size and additional confounders to further elucidate underlying factors of reduced PF and mobility after PBO.

